# The effect of cardiac catheterization on thyroid functions in infants with congenital heart diseases: a prospective observational study

**DOI:** 10.1007/s00431-024-05934-4

**Published:** 2025-01-15

**Authors:** Boshra A. Elbaz, Hala M. Elmarsafawy, Wafaa N. Laimon

**Affiliations:** 1 Department of Pediatrics, Neonatology Unit, Mansoura New General Hospital, Mansoura, Egypt; 2 Pediatric Cardiology Unit, Department of Pediatrics, Mansoura Faculty of Medicine, Mansoura University, Mansoura, Egypt; 3 Faculty of Medicine, New Mansoura University, New Mansoura, Egypt; 4 Pediatric Endocrinology and Diabetes Unit, Department of Pediatrics, Mansoura Faculty of Medicine, Mansoura University Children’s Hospital, Mansoura University, Gomhoria Street, Mansoura, 35516 Dakhlia Egypt

**Keywords:** Hypothyroidism, Congenital heart diseases, Iodinated contrast media, Cardiac catheter, Fluoroscopy, Thyroid functions

## Abstract

This study aims to determine the incidence, clinical course, and risk factors of hypothyroidism following cardiac catheter (CC) in infants with congenital heart diseases (CHD). This prospective study involved 115 patients with CHD, all aged 3 years or younger, who underwent CC, as well as 100 healthy age- and sex-matched controls. Baseline thyroid function tests (TFTs) were conducted for both the patients and controls. In the CHD cohort, TFTs were reassessed 4 weeks after the CC, and for those with abnormal TFT values at this time, the tests were repeated after 2 weeks. Levothyroxine was started for patients with persistent abnormal TFTs, at 4 weeks and 6 weeks assessments after CC. Four weeks after CC, 12% of the studied group exhibited hypothyroidism. Univariate analysis identified significant predictors of hypothyroidism following CC: aortic stenosis (*RR* = 10.0 (1.49–66.99), *P* = 0.018), duration of fluoroscopy during CC (*RR* = 2.12 (0.99–4.26), *P* = 0.05), and total iodinated contrast media (iCM) during CC (*RR* = 2.5 (1.35–3.55), *P* = 0.019). Multivariate analysis indicated that iCM dose was the sole significant predictor of developing hypothyroidism (*RR* = 2.10 (1.01–3.23), *P* = 0.04). ROC curve analysis showed that the cut-off point of iCM dose for prediction of hypothyroidism evolution is 8.7 gm/kg, (sensitivity: 83%, specificity: 65%), while the cut-off point of fluoroscopy duration which predicts the development of hypothyroidism is 24 min (sensitivity: 83%, specificity: 66%). Acquired hypothyroidism after CC persisted in 4% of this cohort for 6 months.

*Conclusion*: Higher doses of iCM and longer duration of fluoroscope during CC are risk factors for the evolution of hypothyroidism post-CC. We recommend assessing thyroid profile 4 weeks after CC, particularly in patients who received an iCM dose greater than 8.7 gm/kg and/or exposed to fluoroscopy for more than 24 min.

**Table:** 

What is known:
• The use of excess iodine leads to transient inhibition of thyroid hormones biosynthesis via the Wolff-Chaikoff effect.
• Infants with congenital heart diseases (CHD) are more prone to hypothyroidism due to higher frequency of abnormal thyroid morphology and routine exposure to supraphysiological doses of iodine.
What is new:
• Exposure to a total dose of iodinated contrast media more than 8.7 gm/kg and a fluoroscopy duration more than 24 min during cardiac catheter are risk factors for the evolution of thyroid hypofunction following cardiac catheter.

## Introduction

Thyroid hormone is crucial for brain development and functions in young infants, and even transient hypothyroidism in this age group can lead to neurocognitive deficits [1].

The use of excess iodine leads to transient inhibition of thyroid hormones biosynthesis via the Wolff-Chaikoff effect, but the thyroid gland can escape this inhibitory effect of the high intra-glandular iodine after 48 h via auto-release mechanisms. However, the immaturity of this escape phenomenon in young infants renders them more vulnerable to develop hypothyroidism following exposure to large doses of iodine [2–4]. Also, using ionizing radiation even at low doses increases the risk of thyroid hypofunction [5].

Infants with congenital heart diseases (CHD) are more prone to hypothyroidism due to the higher frequency of abnormal thyroid morphology and routine exposure to supraphysiological doses of iodine [3]. The sources of excess iodine in those infants are either iodinated contrast media (iCM) during computed tomography angiography (CT-A) and cardiac catheterization (CC) or topical iodine-containing antiseptics used for disinfection [2, 4]. Furthermore, infants undergoing CC are exposed to variable doses of ionizing radiation which are usually higher during complex procedures [6].

Hypothyroidism can lead to left ventricular hypertrophy, diastolic dysfunction, increased systemic vascular resistance, and reduced cardiac contractility [3].

Although normal thyroid hormone levels are fundamental for proper cardiac functions, thyroid hormonal assay is not routinely monitored in CHD patients undergoing CC. Few studies investigated the frequency of hypothyroidism following iodine exposure during CC in patients with CHD, [2, 7, 8] but with no long-term follow-up data. A single study followed up the patients for a median period of 3 years after CC and concluded that the frequency of acquired hypothyroidism after iodine overload was 15.4% and that it was transient in most patients [4]. None of the published studies assessed the effect of ionizing radiation exposure during CC on the evolution of hypothyroidism in infants with CHD.

The aim of the current research work is to determine the incidence, potential onset, and clinical course of hypothyroidism following CC in CHD patients aged ≤ 3 years and to evaluate the predictors for the development of thyroid hypofunction in this vulnerable group.

## Subjects and methods

### Participants

This prospective study included all patients ≤ 3 years of age with CHD who underwent CC in Mansoura University Children Hospital (MUCH), Mansoura, Egypt, from August 2018 to February 2020. Patients with a pre-existing thyroid dysfunction, genetic disorders, midline facial defects, or renal impairment were excluded.

### Methods

Patients’ data (gender, gestational age, birth weight, age at diagnosis of CHD, type of CHD, age at time of CC, number of procedures using iCM, maximal serum creatinine during exposure duration, total cumulative dose of iCM, and duration of fluoroscopy during CC) were collected. Catheter Risk Score for Paediatrics (CRISP), which predicts the serious adverse events for individuals undergoing CC, was calculated for all patients [9].

Serum thyroid stimulating hormone (TSH) and free thyroxine (FT4) were measured for all patients using chemiluminescent immunoassay (ElectroChemiLuminescence [ECL], Roche Cobas 6000 analyser (F. Hoffmann-La Roche Ltd. Investor Relations CH-4070 Basel Switzerland); at the following time points: before CC and then, 1 day, 1 week, 2 weeks, and 4 weeks after the procedure. These time points were determined depending on the physiological response of the thyroid gland to excess iodine. Hypothyroidism was defined as serum TSH > 10 with or without low serum FT4 [10]. For those with abnormal TFT values 4 weeks after CC, the tests were repeated after 2 weeks. Levothyroxine was started for patients with persistent abnormal TFTs, at 4 and 6 weeks assessment after CC.

In the current study, an iodine-free antiseptic agent (Kodan®Tinktur forte, Schülke & Mayr, Norderstedt, Germany) was used in skin disinfection. In our institution, Omnipaque™ 300 mg I/ml (manufactured by GE Healthcare Ireland, Cork, Ireland) is used as the standard in CT-A and CC. One milliliter of Omnipaque™ 300 contains 647 mg of iohexol (corresponding to 300 mg of iodine) in aqueous solution.

### Ethics

This study was approved by the institutional review board of the Mansoura faculty of medicine and was in accordance with the 1964 Helsinki Declaration and its later amendments. Code number is MS.18.08.233. Informed written consents were obtained from the patients’ parents to conduct and publish this research.

### Statistical analysis

Data were analyzed using the Statistical Package of Social Science (SPSS) program for Windows (Standard version 21). The normality of data was first tested with a one-sample Kolmogorov–Smirnov test. Continuous variables were presented as mean ± SD (standard deviation) for parametric data and median (range) for non-parametric data. Qualitative data were described using number and percent. Association between categorical variables was tested using the Chi-square test, while the Fischer exact test and Monte Carlo test were used when the expected cell count less than 5. A Mann–Whitney U test was used to compare 2 independent groups. Significant predictors in the univariate analysis were entered into the regression model using the forward Wald method /Enter. Adjusted relative risk and their 95% confidence interval were calculated. Receiver operating characteristic (ROC) curve analysis was carried out to determine the cut-off points of total iCM and duration of fluoroscopy for detection of hypothyroidism following CC. For all the above-mentioned statistical tests done, the threshold of significance is fixed at a 5% level (*P-*value). The results were considered non-significant when the probability of error is more than 5% (*P* > 0.05) and significant when the probability of error is equal or less than 5% (*P* ≤ 0.05).

## Results

A total of 128 patients with CHD who underwent CC were enrolled in this study. Thirteen patients were excluded due to pre-existing thyroid hypofunction and/or genetic disorders. A total of 115 participants were included: 47 males (41%) and 68 females (59%), with a median gestational age of 38 (range: 34–40) weeks, and median birth weight of 3 (range: 1.5–4) kilograms. A total of 88% of the study cohort had congenital acyanotic heart diseases. The median age of diagnosis of CHD was 4 (range: 0.07–30) months, and the median age at the time of assessment of thyroid functions was 13.8 (range: 1–36) months.

In the studied cohort, only 12 patients (10%) underwent diagnostic CC, while 103 patients (90%) underwent interventional CC. The indications of the diagnostic catheter in our cohort included assessment of pulmonary artery pressure in 10 patients with large left to right shunt and postoperative evaluation of modified Blalock-Taussig Shunt (MBTS) in 2 patients with pulmonary atresia.

In the studied group, the median cumulative dose of iCM was 5.29 (range: 0.75–15) gm/kg, and the median duration of fluoroscopy exposure was 20 (range: 5–44) min. Patients’ characteristics are summarized in Table 1.

**Table 1:** Characteristics of the study subjects

	Patients (*N* = 115)	Controls (*N* = 100)	Test of significance	*P-*value
Gestational age (weeks), mean ± SD	39 ± 2.34	38 ± 1.70	*t* = 0.186	0.74
Birth weight (kg), median (range)	3.0 (1.93–4.0)	3.2 (2.19–4.20)	*Z* = 1.84	0.066
Gender				
• Male, *n* (%) • Female, *n* (%)	47 (41%) 68 (59%)	40 (40%) 60 (60%)	*χ*2 = 1.77	0.183
Age at time of evaluation (months), median (range)	13.8 (1.0–36.0)	12 (1.5–36)	*Z* = 1.29	0.195
Basline serum TSH (mU/L), mean ± SD	4.15 ± 2.62	3.45 ± 1.75	*t* = 1.14	0.262
Baseline serum free T4 (pmol/L), mean ± SD	11.10 ± 2.62	12.15 ± 3.21	*t* = 1.55	0.129
Age at diagnosis of congenital heart disease (CHD) (months), median (range)	4.0 (0.07–30.0)	-	-	-
Types of CHD				
• Left to right shunt, *n* (%) • Obstructive lesions, *n* (%) • Cyanotic, *n* (%)	68 (59%) 33 (29%) 14 (12%)			
CRISP^¶^, median (range)	5.0 (0.0–13.0)	-	-	-
Number of procedures with iodinated contrast media exposure, median (range)	1.0 (1.0–4.0)	-	-	-
• One procedure, *n* (%) • Two procedures, *n* (%) • Three procedures, *n* (%) • Four procedures, *n* (%)	90 (78%) 14 (12%) 7 (6%) 4 (4%)	-	-	-
Types of cardiac catheters				
• Diagnostic catheter, *n* (%) • Therapeutic catheter, *n* (%)	12 (10%) 103 (90%)			
Maximum serum creatinine during exposure period (mg/dl), median (range)	0.51 (0.30–0.80)	-	-	-
Maximum serum blood urea nitrogen during exposure period (mmol/L), median (range)	4.7 (1.8–6.4)	-	-	-
Total iodine dose used during cardiac catheter at index date (gram per kg)	5.29 (0.75–15.0)	-	-	-
Duration of fluoroscopy exposure during cardiac catheter at index date (minutes), median (range)	20.0 (5.0–44.0)	-	-	-

*χ*^2^, Chi-square test; *t*, Student’s *t*-test; *Z*, Mann–Whitney U test

*¶CRISP*, catheterization risk score for pediatrics

¶¶Any procedure included exposure to iodinated contrast media (surgical and/or radiological)

**Fig. 1:**
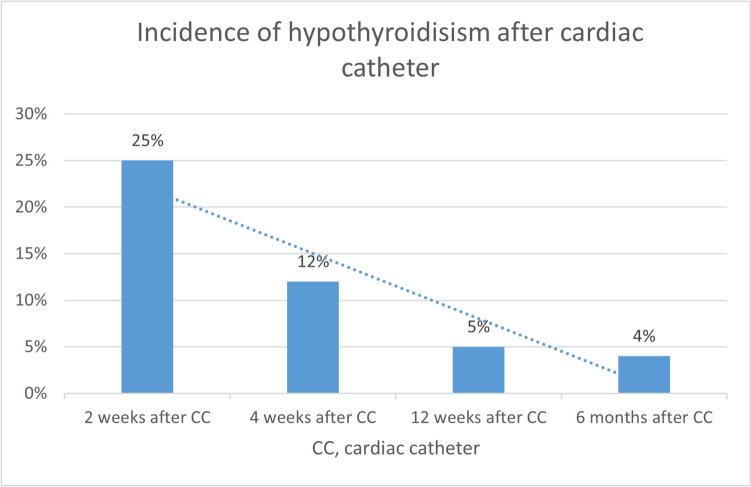
The incidence of hypothyroidism in the studied cohort at different time intervals following the cardiac catheter

Thyroid function tests showed thyroid hypofunction in 24% of patients (2 weeks after CC) versus 12% of patients (4 weeks after CC) (Fig. 1). Patients with abnormal thyroid profile 1 month after CC showed neither clinical evidence of hypothyroidism nor thyroid abnormalities in ultrasound examination.

All patients with SH 4 weeks post-cardiac catheterization were assigned into one group “hypothyroidism group” (n = 14) and were compared with patients with normal thyroid profile (*n* = 101). This comparison revealed that aortic stenosis frequency was higher in the hypothyroidism group (50%) as compared to the euthyroid group (8.8%), *P* = 0.029. Patients who developed hypothyroidism have received significantly higher cumulative dose of iCM, *P* = 0.007, and they had a significant longer fluoroscopy exposure time,* P* = 0.026, when compared with the euthyroid group (Table 2).

**Table 2:** Relation between thyroid functions and patients’ characteristics

	After 4 weeks		Test of significance	*p-*value
	Euthyroid (*n* = 101)	Hypothyroidism (*n* = 14)		
CRISP¶, median (range)	5.0 (0.0–13.0)	5.0 (3.0–12.0)	*Z* = 0.798	0.425
Type of congenital heart disease				
• Left to right shunts, *n* (%) -Atrioventricular septal defect -Ventricular septal defect -Atrial septal defect -Patent ductus arteriosus • Obstructive, *n* (%) -Pulmonary stenosis -Aortic stenosis • Cyanotic, *n* (%) -Transposition of great arteries	8 (8) 9 (9) 15 (15) 51 (50) 8 (8) 10 (10) 0 (0.0)	0 (0.0) 0 (0.0) 0 (0.0) 4 (29) 2 (14) 7 (50) 1 (7)	FET FET FET FET FET *χ*^2^=1.49 FET	1.0 0.1 0.221 0.395 0.643 0.029^*^ 0.12
Number of procedures¶¶, median (range)				
• One procedure, *n* (%) • Two procedures, *n* (%) • Three procedures, *n* (%) • Four procedures, *n* (%)	78 (78) 11 (11) 7 (7) 4 (4)	12 (86) 2 (14) 0 (0.0) 0 (0.0)	MC	0.878
Types of cardiac catheters				
• Diagnostic catheter, *n* (%) • Therapeutic catheter, *n* (%)	12 (12) 89 (88)	0 (0.0) 14 (100.0)	FET	1.0
Duration of fluoroscopy exposure during cardiac catheter (minutes), median (range)	19.5 (5.0–44.0)	28.0 (20.0–35.0)	*Z* = 2.23	0.026^*^
Total iodine dose used during cardiac catheter (gram per kg)	5 (0.75–15.0)	11 (8.11–15.0)	*Z* = 2.61	0.007^*^

*χ*^2^, Chi-square test; *Z*, Mann–Whitney U test; *FET*, Fischer’s exact test; MC, Monte Carlo test; *Significant *p* value < 0.05

¶CRISP, catheterization risk score for pediatrics

¶¶All procedures which included exposure to iodinated contrast media (surgical and/or radiological)

Univariate analysis showed that the significant predictors of evolution of hypothyroidism following CC are aortic stenosis (*RR* = 10.0 (1.49–66.99), *P* = 0.018), duration of fluoroscopy (*RR* = 1.12 (0.99–1.26), *P* = 0.05), and total cumulative dose of iCM (*RR* = 1.01 (1.003–1.01), *P* = 0.019). Multivariate analysis revealed that dose of iCM was the only significant predictor of developing hypothyroidism in the studied cohort (*RR* = 1.00 (1.00–1.01), *P* = 0.04) (Table 3).

**Table 3:** Univariate and multivariate analysis of predictors of hypothyroidism among the studied patients

Predictors	Univariate analysis		Multivariate analysis	
	*P*	RR (95% CI)	*P*	RR (95% CI)
Type of cardiac lesion:				
Aortic stenosis	0.018^*^	10.0 (1.49–66.99)	0.256	3.76 (0.38–37.13)
Duration of fluoroscopy exposure during cardiac catheter	0.05^*^	2.12 (0.99–4.26)	0.121	1.31 (0.97–2.54)
Total iodine dose used during cardiac catheter	0.019^*^	2.5 (1.35 −3.55)	0.04^*^	2.10 (1.01–3.23)

*RR*, relative risk; *CI*, confidence interval

^*^Significant *p-*value ≤ 0.05

**Fig. 2:**
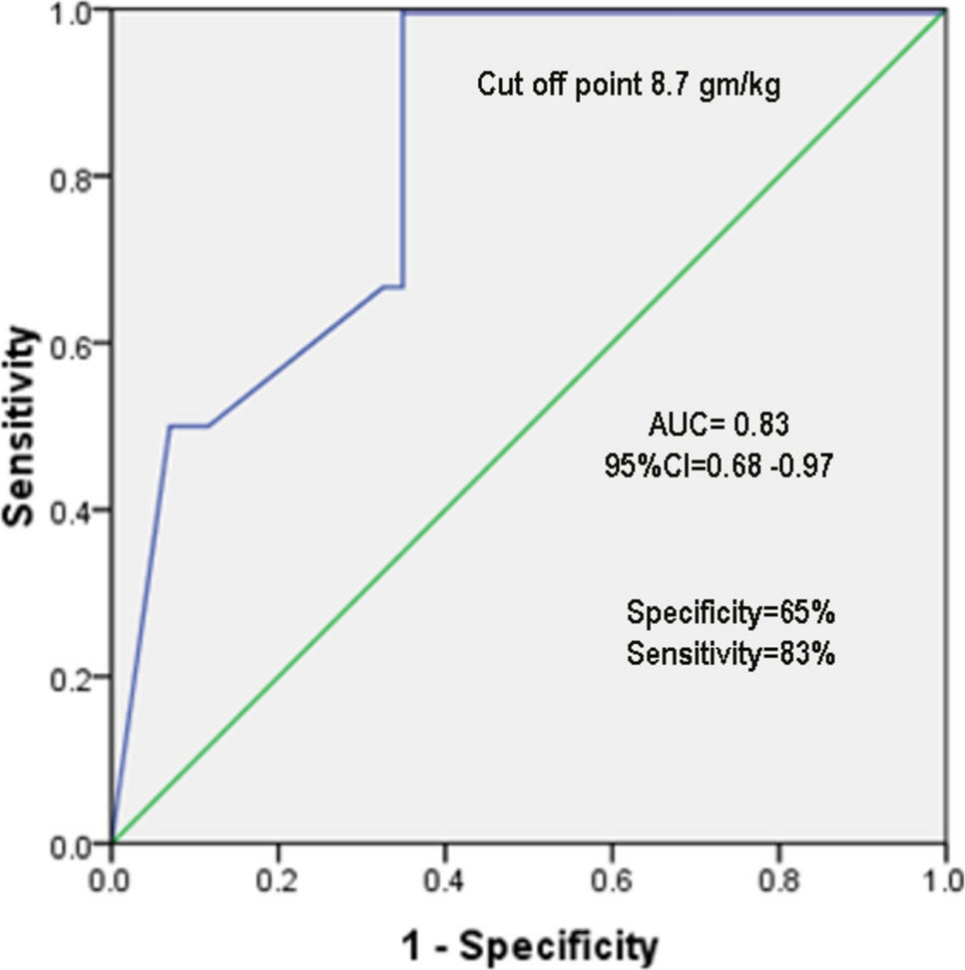
ROC curve for iodine dose which can predispose to hypothyroidism among the studied cases

**Fig. 3:**
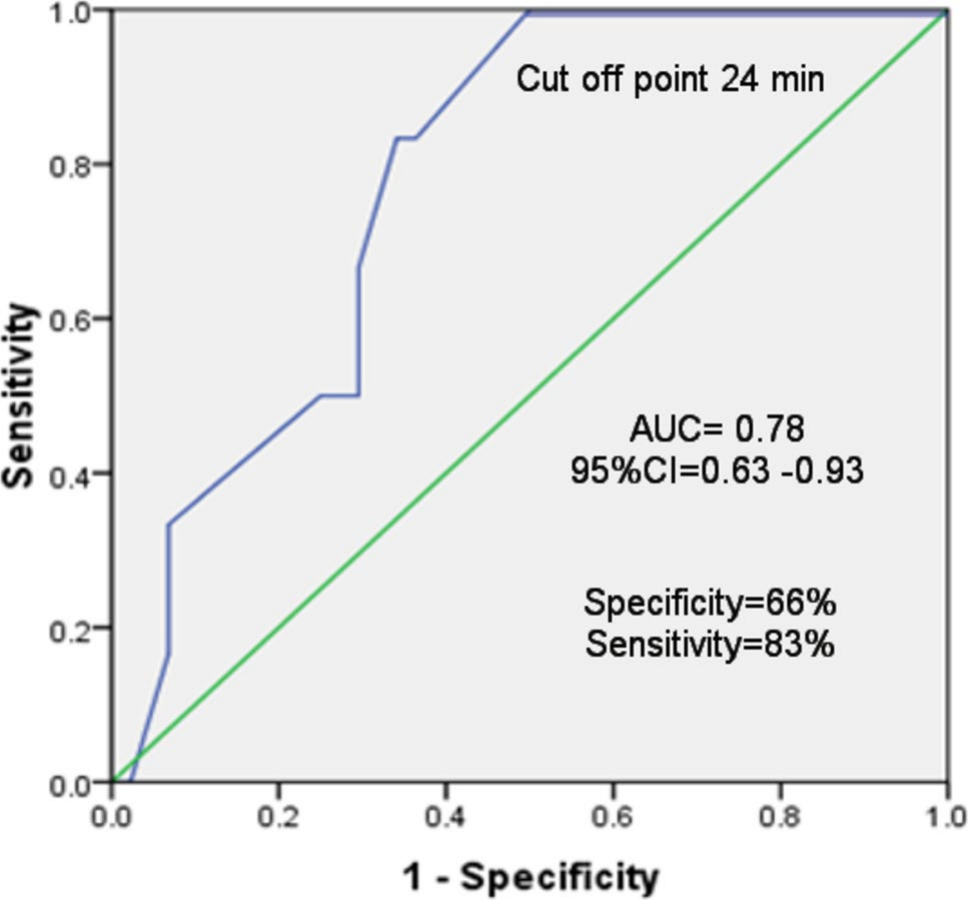
ROC curve for fluoroscopy duration which can predispose to hypothyroidism among the studied cases

ROC curve analysis has shown that the cut-off point of cumulative dose of iCM for prediction of developing thyroid hypofunction post cardiac catheterization is 8.7 gm/kg, with a sensitivity of 83.3% and a specificity of 65.1% (Fig. 2). In our cohort, the cut-off point of fluoroscopy duration which predicts evolution of hypothyroidism following CC is 24 min, with a sensitivity of 83.3% and a specificity of 65.9% (Fig. 3).

Levothyroxine was started for twelve patients with SH. During a mean follow-up duration of 2.25 years, levothyroxine was successfully discontinued in seven patients (58%) after a mean duration of 6 months. 5/12 patients (42%) continued to receive long-term levothyroxine for a median period of 20 months (range: 6–36 months).

## Discussion

Recent years have seen rapid development of CC as a diagnostic and a therapeutic modality for management of CHD [11]. However, little data are available about the potential hazards of exposure to excess iodine and ionizing radiation during CC on the thyroid functions in patient with CHD.

Treatment of SH is still a matter of debate. However, the beneficial effect of levothyroxine therapy on neurocognitive outcomes and reducing cardiometabolic risk factors has been reported [12].

The current prospective study aimed to assess post-cardiac catheter thyroid functions in CHD patients, aged ≤ 3 years. In this study, the incidence of hypothyroidism 1 month after CC was 12%. However, seven out of these twelve patients had transient thyroid hypofunction. This finding is consistent with previous reports of thyroid dysfunction following CC [2, 4, 7, 8]. In a retrospective study conducted on 104 children with CHD (aged from 0–8 years), post-cardiac catheter hypothyroidism was reported in 15.4% of the studied population and it was transient in 14 out of 16 patients [4]. Thaker et al. [2] and Dechant et al. [8] reported that the incidence of thyroid hypofunction following CC was 25% and 28.5%, respectively.

The mechanism of hypothyroidism following CC is multifactorial. Excess iodine blocks the sodium iodine co-transporter and inhibits thyroglobulin synthesis (Wolff–Chaikoff effect) [13]. The mature thyroid gland usually escapes this inhibitory effect of iodine within a few days. However, repeated exposure to large doses of iodine particularly in young infants leads to failure of this escape phenomenon [2]. In addition to the injected iodine during different procedures, a large amount of topical iodine is applied for disinfection, but it is not practically possible to assess its quantity. Moreover, low-dose ionizing radiation exposure seems to increase the risk of hypothyroidism [5]. Infants undergoing complex procedures during CC are expected to be exposed to longer durations and larger doses of ionizing radiation.

In previous studies, cumulative dose of iCM, number of interventional procedures, intake of drugs affecting thyroid functions (e.g., amiodarone), impaired renal functions, and intensive care unit stay were identified as risk factors for the evolution of hypothyroidism in infants with CHD [2, 7]. In the current study, larger total iCM and longer duration of fluoroscopy were found to be risk factors for developing thyroid hypofunction following CC. Furthermore, this research work identified a cumulative iCM dose of 8.7 gm/kg and a duration of fluoroscopy of 24 min as predictors of developing SH after CC with a sensitivity of 83% and specificity of 65%.

To the best of our knowledge, this is the largest prospective study from a single center which evaluates thyroid functions following CC in CHD patients younger than 3 years with the exclusion of genetic disorders which can be associated with thyroid abnormalities. Serial assessment of thyroid function tests adds to the strength of our study. Moreover, this is the first study to assess the effect of fluoroscopy duration during CC on thyroid profile.

The current study is limited by being a single-center study and lack of assessment of renal elimination of iCM as urinary iodine was not measured.

Further multicenter studies with larger sample sizes would be better positioned to study the effect of CC on thyroid functions.

## Conclusion

The incidence of hypothyroidism in infants with CHD 4 weeks following CC is 12%. Assessment of thyroid function is not routinely performed for infants undergoing CC; hence, the true incidence of SH in this vulnerable group is likely to be more than the estimated incidence in the current study and previous research work. Exposure to a higher dose of iCM and a longer duration of fluoroscope during CC are risk factors for the evolution of thyroid hypofunction. In the light of these study results, we recommend assessment of thyroid profile 4 weeks after CC, particularly in patients who received a dose of iCM higher than 8.7 gm/kg and/or exposed to fluoroscope for more than 24 min.

## Data Availability

Data cannot be shared openly, to protect study participant privacy. However, they can be shared by the corresponding author with a reasonable request.

## Abbreviations

CHD: Congenital heart diseases
iCM: Iodinated contrast media
CT-A: Computed tomography angiography
CC: Cardiac catheterization
TSH: Thyroid stimulating hormone
FT4: Free thyroxine
ECL: Electrochemiluminescence
ROC: Receiver operating characteristic
SH: Subclinical hypothyroidism

## Code availability

The clinical data were analyzed using the Statistical Package of Social Science (SPSS) program for Windows (Standard version 21).
